# Analysis on the Performance of Micro and Nano Molybdenum Di-Sulphide Powder Suspended Dielectric in the Electrical Discharge Machining Process—A Comparison

**DOI:** 10.3390/nano12203587

**Published:** 2022-10-13

**Authors:** Rajesh J. V., Giridharan Abimannan

**Affiliations:** School of Mechanical Engineering, Vellore Institute of Technology Chennai, Chennai 600127, Tamil Nadu, India

**Keywords:** molybdenum-di-sulphide, micro and nano powder, powder mixed electrical discharge machining, crater geometry, modelling, deposition, surface crack density, skewness and kurtosis

## Abstract

The significance of suspending molybdenum di-sulphide powder particles of two distinct mean size viz. Φ40 μm and Φ90 nm into the dielectric of electrical discharge machining is analysed. Crater geometry, surface crack density, skewness, kurtosis and chemical alteration of machined surfaces are considered as outcome measures. A numerical model using finite element analysis is developed to forecast crater geometry. To validate the proposed model, experiments are conducted by varying input parameters such as discharge duration, peak current, and gap voltage. In comparison with the experimental results, the proposed model predicts diameter of crater with an error of 3.34%, 7.32% and 2.76% for discharge duration, peak current and gap voltage respectively for Φ40 μm powder; similarly, 0.19%, 3.65% and 2.78% for Φ90 nm powder. Scanning electron microscope images, 2D roughness profiles and X-ray diffraction profiles are used to assess the partial discharge phenomena, surface crack density, skewness, kurtosis and chemical alteration of the machined surface. For all parameter settings, the Φ90 nm produced surfaces with lessened micro-cracks compared to Φ40 μm. The Φ90 nm creates surfaces with negative skewness and kurtosis less than 3. The deposition of MoS_2_ powder particle on the machined surface is revealed through X-ray diffraction analysis.

## 1. Introduction

Electrical discharge machining (EDM) is a widely accepted non-traditional subtractive machining technique employed to create intricate contours/forms on difficult-to-cut materials used in aerospace, automotive, marine, dies and mould-making industries [1,2]. Material removal in the EDM process occurs due to electro-thermal spark erosion phenomena [3,4]. In the EDM process, the tool and workpiece (hereafter ‘electrodes’) are connected to a high-frequency pulse generator. The electrodes are kept immersed inside a dielectric liquid (mostly hydrocarbon-based liquid) separated by a small distance called a ‘spark gap’. When the power is switched on, the pulse generator supplies high-frequency pulses between the electrodes. As the pulses occur in the gap, the insulating property of the dielectric medium is broken transitorily, which allows a discharge (spark) to occur between the electrodes. As the discharge remains, continuous melting, evaporation of the dielectric liquid and electrode material takes place. Owing to electro-thermal heating, a molten pool and a compressed vapour bubble is formed on the electrode’s surface and around the discharge respectively. The instant termination of electric pulse collapses the compressed vapour bubble, as a consequence the bubble bursts abruptly. As a result, a portion of the melted material present on the electrode’s surface is expelled into the dielectric medium and the remainder is re-deposited back onto the adjacent region. Therefore, each pulse removes a minuscule volume of material from the electrode’s surface which results in a crater forming [3,4,5]. Due to its tendency for reduced material removal, various techniques have been employed to improve the existing EDM process without sacrificing its inherent characteristics. In the past, methods such as applying tool electrode rotation [6], ultrasonic vibration to workpiece or tool material [7], use of powder metallurgical tools [8] and suspending fine powder particles into the dielectric fluid with continuous stirring [5] were employed. Among these, suspending fine powder particles (metals, semi-conductor and ceramic) into the liquid dielectric, known as powder mixed EDM (PMEDM), has gained researchers’ attention as it offers improved performance under the same operating conditions [9,10]. The process mechanics of PMEDM is identical to EDM except the dispersion of fine powder particles into the liquid dielectric. The suspended powder particles reduce the insulating strength of the dielectric. These powder particles act as secondary sites of discharges and enhances the discharge characteristics. Each secondary discharge produces a crater over the work surface [10,11,12,13,14,15]. The performance of the EDM or PMEDM process is entirely dependent on the crater morphology. Therefore, understanding the formation of craters on any machined surface is mandatory as it demonstrates the EDM or PMEDM process characteristics.

Several disciplines such as electric, thermal, magnetic, hydraulic, and dynamics involved in EDM process which make process modelling a complex stochastic phenomenon [16]. Several researchers have made significant contributions to the development of models to mimic crater formation phenomenon in both EDM and PMEDM process. Snoeys et al. [17] presented a thermo-mathematical model considering a 2D heat flow semi-infinite cylinder with a disc heat source model, assuming the energy provided to the workpiece is 50% and the heat source existed during the pulse alone. This was extended by Van Dijck and Dutre [18] for two distinct continuums (finite and infinite). Jilani and Pandey [19] presented a model to forecast plasma channel growth assuming that the plasma channel propagates with the time by considering discharge energy and electrode properties. DiBitonto et al. [20] and Patel et al. [21] proposed a theoretical model of cathode erosion using a point heat source and the energy distribution to the cathode is assumed to be 18%. Later Joshi and Pande [3] developed a “thermo-physical model” to predict the crater diameter using the finite element analysis. The analysis is carried out by considering “more realistic assumptions such as gaussian heat distribution of heat flux and spark radius equation based on discharge current and discharge duration”. Compared to DiBitonto’s work, the model developed by Joshi and Pande [3] gave better prediction of crater geometry. However, the model is validated with limited experimental data and also failed to expose for wide range of input parameters due to difficulty to incorporate real-life conditions in the analysis. Tang and Yang [22] established a unique “thermal-hydraulic coupling model” to investigate the morphology of crater in EDM process using a level-set method. Tao et al. [23] proposed a crater formation model using the fluid dynamics program FLUENT considering the plasma heating and bubble collapsing phase. Authors proposed that the crater depth is dependent on the specific volume of the solid metal, i.e., the expansion of the material volume. Giridharan and Samuel [24] proposed a model to predict carter depth using 2D surface roughness profiles. George et al. [25] used the time integration effect (TIE) approach to figure out the crater’s radius and depth by taking into account distinct plasma flushing efficiencies of 95% and 45% for carter diameter and crater depth, respectively, during the WEDT process. Yeo et al. [26] provided a comprehensive numerical comparison of electro-thermal EDM models. Shabgard et al. [27,28] proposed a model to forecast crater dimensions during the EDM process and explored the impact of plasma flushing efficiency (PFE) in the EDM process, stating that PFE is a function of discharge current and time. However, the proposed PFE equation is valid for small magnitudes of pulse duration and discharge current only. Jithin et al. [29] introduced the variable PFE factor in the heat flux equation to increase the prediction accuracy of the crater profile/shape during the EDM process.

Authors have made an effort to analyse the crater morphology formed during the PMEDM process with different powder particles. Tzeng et al. [14] examined the influence of powder properties such as thermal conductivity, electrical resistivity, particle size, and concentration on machining efficiency. Rajeswari and Shunmugam [30] conducted a thorough investigation by suspending graphite powders (10 µm size) and induced ultrasonic vibration into the dielectric medium and studied the variation in EDM gap phenomena using pulse train analysis. The authors stated that use of ultrasonic vibration in the PMEDM process helps to eject debris effectively from the discharge gap and prevents agglomeration between the suspended powder particles. So far, several authors have carried out experimental investigations using different powder particles such as Gr, Si, SiC, Ti, CNT, MWCNT, Cu, Al, Fe, and molybdenum-di-sulphide (MoS_2_) on various workpiece materials to assess the crater morphology and performance of the machined surface in the PMEDM process [7,8,9,10,11]. Ekmekci et al. [31] proposed a postulate-on-discharge separation model to understand the crater formation in the PMEDM process. Kansal et al. [5] suggested a model by introducing the term ‘spark frequency factor’ to account for the influence of powder characteristics suspended into the dielectric medium during the PMEDM process. A constant value of 2.4 is used as spark frequency factor for simulation and validated with limited experimental runs. Furthermore, the usage of spark frequency factor for wide range of process parameter conditions is yet to be explored. Bhattacharya et al. [15] and Singh et al. [16] presented a FEM model to estimate the diameter and depth of crater formed during PMEDM process considering multiple discharge phenomena. The authors proposed that the pulse on-time and current have a significant effect on crater morphology. Later, Zhao et al. [32] developed a model using joule heating to analyse the heat conducted into a work surface in a single discharge of the PMEDM process. Authors postulated that the heat flux incident on the workpiece is influenced mostly by current, voltage, and spark radius magnitudes. Vijaykumar et al. [33] developed a 3D-axisymmetric model considering single-discharge phenomena [34,35] during the PMEDM process. The authors investigated characteristics such as surface crack density, skewness and kurtosis of machined surfaces to assess the functional performance of the components for various applications [36,37,38,39,40,41]. Arun Kumar et al. [37] found that the magnetic field-assisted PMEDM process produces fewer surface cracks than normal EDM process and postulated that the discharge current and discharge duration are the most influencing parameters. Govindan and Joshi [38] addressed the crack formation mechanism in dry EDM and liquid dielectric EDM process. The authors identified that the average length and density of micro-cracks were lessened in the dry EDM compared to conventional EDM. Coelho and Koshy [40] attempted to explore the possibility of using EDM-machined inserts for passive vibration damping applications. It was reported that the damping ratio of EDM-machined inserts is doubled compared to the extruded insert due to the positive skewness and high kurtosis produced on machined surfaces. Tran et al. [41] employed the Taguchi technique and ANOVA analysis for improving the surface finish of 90 CrSi steel by suspending SiC powder particles in dielectric liquid of the EDM process. Input parameters such as powder concentration, pulse-on-time, pulse-off-time, pulse current, and servo voltage were used. The authors reported that the powder concentration and pulse-off-time showed a significant effect on surface finish compared to other parameters. Rehman et al. [42] investigated the effect of adding different-sized graphite powder particles (20, 30 and 40 μm) to the dielectric liquid during the EDM process; the Box–Behnken design was used to predict the machining characteristics of EN-30B alloy steel. The authors postulated that input factors such as peak current, pulse-on-time, the size and concentration of the graphite powder had a substantial impact on the recast layer thickness, micro hardness, crater size, MRR, and TWR, which are considered as output measures. At higher concentrations and larger particle sizes, the crater depth and micro hardness were maximized. Nguyen et al. [43,44] applied grey relation analysis (GRA) to identify the optimum machine settings to achieve minimum surface roughness, electrode wear rate and maximum material removal rate in SiC-suspended dielectric liquid of the EDM process. Alhodaib et al. [45] attempted to identify optimum settings using GRA in machining Nimonic 90 superalloy and various other materials [46]. Table 1 shows a critical review of existing works and the novelty of the present study.

Based on the critical observations identified from the existing literature (as presented in Table 1), the present study aims at developing a single-spark transient thermal model for the PMEDM process to predict the crater geometry (diameter and depth). A new parameter called “variable pulse frequency factor” is included to account the addition of powder into the dielectric medium. The proposed model is verified by conducting PMEDM experiments covering full range of parameters used in the industry. In order to explore the performance of nano-sized MoS_2_ powder (mean size Φ90 nm) particles, micron-sized MoS_2_ powder (means size Φ40 μm) particles were chosen for comparison. The significance of the input process parameters, namely, discharge duration, peak current, gap voltage and powder particle size on surface characteristics such as crater morphology, surfaces crack density, skewness, kurtosis and chemical alteration of the machined surface, are analysed. The scanning electron microscope (SEM) images, 2D roughness profiles, energy dispersive spectroscopy (EDS) and X-ray diffraction (XRD) profiles are used to assess the surface characteristics. Details of the models, experimentation and obtained findings are presented in subsequent section of this paper. 

### 1.1. Modeling of PMEDM Process

In the PMEDM process, the assumptions considered in the development of a thermal model to predict crater geometry are listed below.

(a) Every pulse is assumed to be a spark pulse and the influence of inactive (short and open voltage) pulses are neglected.
(b) Each discharge pulse generates a crater on the workpiece.
(c) The discharge channel is assumed to be perfectly conductive [50]. The energy transferred by partial discharge is insignificant compared to primary discharge. Hence the effect of partial discharges occurred due to suspension of powder particles are neglected.
(d) A Gaussian distribution-shaped heat source is considered and the occurrence of heat flux on the domain is presumed to be axisymmetric [3,5].
(e) During discharge, a fraction of overall input energy is conducted into the workpiece [20]. The convective and radiation transfer coefficients are considered insignificant.
(f) The material is homogeneous, isotropic, and the average thermo-physical properties of work materials are considered constant in all phases [3,20].
(g) The spark radius is a function of the discharge current and discharge duration [3].
(h) During spark discharge, the portion of workpiece material in the boiling region are removed completely. At the end of every discharge, only a fraction of melted material is detached from workpiece surface.
(i) The pulse frequency factor is considered to assess the crater geometry.

A three-dimensional transient heat conduction equation without internal heat generation is considered for the PMEDM process, which is expressed as

(1) $$\frac{1}{r}\;\frac{\partial }{\partial r}\left(\mathrm{kr}\frac{\partial T}{\partial r}\right)+\frac{1}{r^{2}}\frac{\partial T}{\partial \emptyset }\left(k\frac{\partial T}{\partial \emptyset }\right)+\frac{\partial }{\partial z}\left(k\frac{\partial T}{\partial z}\right){=\rho c}_{p}\frac{\partial T}{\partial t}$$

where, r, ∅, and z are the cylindrical coordinates of the work domain. k is thermal conductivity (J/mk), T is the temperature (K), ρ is the density (kg/m^3^), and c_p_ is the specific heat capacity (J/kgK) of the workpiece material. An axisymmetric work domain is considered for this analysis $\frac{\partial T}{\partial \emptyset }=0$. Thus Equation (1) becomes [3,5].

(2) $${\rho \;C}_{p}\frac{\partial T}{\partial t}=\left[\frac{1}{r}\;\frac{\partial }{\partial r}\left(\mathrm{kr}\;\frac{\partial T}{\partial r}\right)+\frac{\partial }{\partial z}\left(k\frac{\partial T}{\partial z}\right)\right]$$

**Figure 1:**
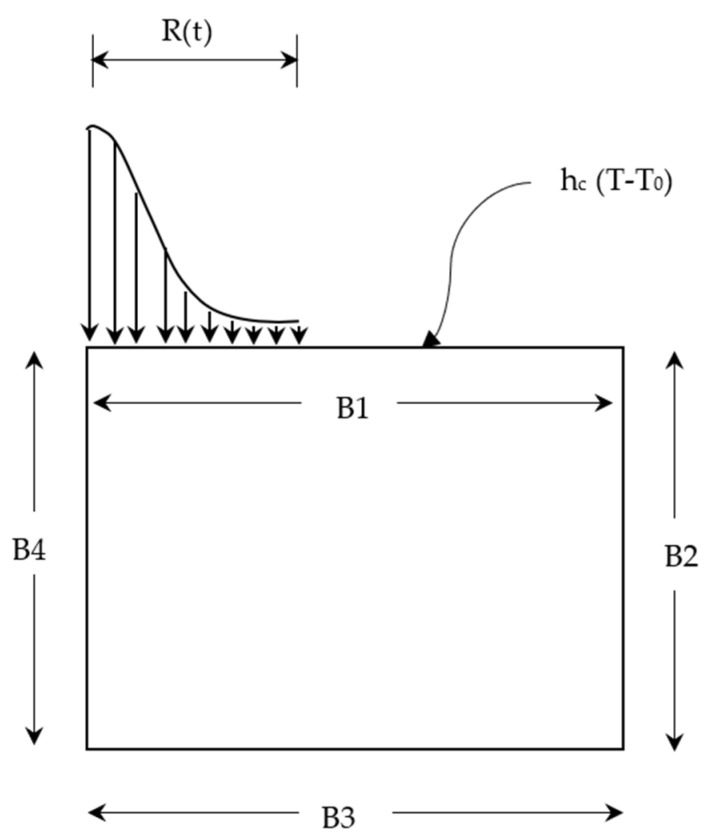
Heat transfer model for PMEDM process.

The domain used in this work is a small cylindrical portion of the workpiece surrounding the spark that is bounded by four boundaries denoted by the letters B1, B2, B3, and B4. Figure 1 depicts the domain and boundary conditions for various workpiece surfaces in the PMEDM process. At the workpiece boundary B1, a single-spark Gaussian heat flux is applied up to the spark radius R, and the remainder of the boundary is assumed to be convective heat transfer between the dielectric medium and the workpiece surface. The boundaries B2 and B3 are positioned away from the source of the spark to ensure no heat is transferred across them. B4 is axisymmetric, hence, the heat flow is assumed to be zero because there is no heat loss or gain in this region. The boundary conditions are given in Equation (3) [3,5].

(3) $$k\left(\frac{\partial T}{\partial z}\right)=\{\begin{array}{cc} {\;h\;}_{c}{(T-T}_{0}) & r\;>\;R\\ Q_{w} & r\;\leq \;R\;\mathrm{on}\;B1\\ 0 & \mathrm{For}\;\mathrm{pulse}\;\mathrm{off}\;\mathrm{time}\; \end{array}$$

and for boundaries B2, B3, B4: $\frac{\partial T}{\partial n}=0$.

Here, h_c_ is the convective coefficient of heat transfer (W/m^2^K), $Q_{w}\left(r\right)$ is the heat flux (W/m^2^) owing to the spark, T_0_ is the room temperature, T_a_ is ambient temperature, T_a_ = T_0_ at t = 0.

Gaussian distribution of heat flux as the heat source model gave better prediction results in comparison to the previous models [3,5,16]. As a result, it has been widely accepted as a heat source for modelling of the PMEDM process. So, the present work considers Gaussian heat flux distributions.

(4) $$Q_{w}(r)=\frac{4{.45\;F}_{c}{\;V\;K}_{n}\;I}{{\pi R}^{2}}\;\exp\left\{-4.5{\left(\frac{r}{R\left(t\right)}\right)}^{2}\right\}$$

where F_c_ is a fraction of heat input to the workpiece, V is discharge voltage (V), I is discharge current (A), R(t) is spark radius (µm), r is the radial distance from the axis of the spark (µm), and K_n_ is pulse frequency factor. It is observed from the pieces of research that the proposed values for the fraction of heat (F_c_) transferred to the workpiece varied from 0.18 to 0.5. DiBitonoto’s group [20,21] stated that a fraction 0.08 and 0.183 of heat is transferred to the anode and cathode respectively. Shankar et al. proposed a hypothesis that 0.4–0.45 of Fc is absorbed by the workpieces [34]. Kansal et al. [5] used 0.05 to 0.2 of F_c_ whereas Battacharya and Batish [15] considered 0.1 to 0.25 during the PMEDM process. In the present model, the value of F_c_ is assumed to be 0.183 and its effects on crater geometry are analysed. In practice, it is very difficult to measure the spark radius experimentally due to the rapid collapse of the plasma channel (order of a few microseconds). To predict the accurate spark radius, many researchers have proposed various approaches. Pandey and Jilani [19] establish the spark radius equation based on the boiling point of the material and had proposed an empirical relation to forecast spark radius for limited tool and work material combination. Later Ikai et al. [35] proposed a semi-empirical equation to determine the spark radius, which is a function of discharge current (16 to 26 A) and discharge duration (100 μs to 700 μs). In this work, the spark radius is evaluated by using the expression below (equivalent heat input radius).

(5) $${R(t)=2040\;\times \;I}_{p}^{\;0.43}{\;\times \;T}_{d}^{\;0.44}$$

During spark discharges, the material in the spark zone melts owing to electro-thermal heating. At the end of discharge, only a portion of melted material is evacuated from the spark zone and the remainder solidifies back onto the adjacent region. In the modelling, a parameter called material removal efficiency or plasma flushing efficiency (PFE) is used to define the entire portion of melted material ejected from the workpiece surface after each spark discharge. Numerous studies have been carried out modelling the EDM by taking into account 100% PFE [2,16,33]. Due to the inherent characteristics of the PMEDM process, it is difficult to attain 100% PFE in practice. Some authors have reported that the PFE of the electrodes varies between 2 and 96% [5,22]. In this present work, the PFE is assumed to be 95% and 45% for crater diameter and depth, respectively [25].

Pulse frequency refers to the number of discharges occurring between the electrodes per unit time, which directly influences the EDM process characteristics. It is perceived from the available literature that, during the PMEDM process, the pulse frequency increases by 2–3 times compared to normal EDM under identical operating conditions [5] because the suspended powder particles in the dielectric medium under the electrostatic forces become polarised in a zig-zag pattern. The dispersed powder particles alter the breakdown characteristic of the dielectric fluid and the interspace for electric discharge initiation is increased (pulse frequency is increased). Earlier, to model the PMEDM process, the authors considered a constant frequency factor (K_n_ = 2.4) [5]. However, in practice, the spark frequency is not constant and depends on various parameters such as discharge duration, peak current, gap voltage, duty factor, powder particle size, powder concentration, particle density, and flushing conditions at the discharge gap. It is an important parameter for inclusion in the heat flux equation to account for the changes that occur in the inter-electrode gap conditions. This makes the model more realistic than existing models. The pulse frequency factor is the ratio of the number of spark pulses in PMEDM to the number of spark pulses in EDM under the same operating conditions. In this work, the spark frequency factor is obtained using the expression below:

(6) $$\mathrm{Pulse}\;\mathrm{frequency}\;\mathrm{factor}\left(K_{n}\right)=\frac{N_{p}}{N}$$

where N_p_—Number of spark pulses acquired in PMEDM and N—Number of spark pulses acquired in EDM.

### 1.2. Steps Involved in FEM Simulation for Crater Formation Using ANSYS

The crater formation steps are carried out by considering the governing Equation (2) along with the boundary conditions mentioned in Equation (3). A 600 μm × 600 μm two-dimensional continuum is employed for FEM analysis. A four-nodded axi-symmetric thermal solid element (PLANE 55) [3] is considered. An ANSYS^TM^ 16.0 parametric design language is used to develop the single spark PMEDM model [52] and repeated for various input conditions [5]. The procedures adopted to generate craters are presented below

1. A 2D model is constructed using the PLANE 55 element with a mesh size of 10 μm.
2. The average thermal and physical properties of workpiece materials, such as k, c_p_, and ρ, are considered.
3. The heat flux Equation (4) applied at the spark radius region as shown in Figure 1.
4. The initial conditions and bulk temperature are set at 298 K. The temperature distribution is determined by the duration of the discharge. The mesh elements and nodes present above the melting point of the workpiece are identified and removed.

**Figure 2:**
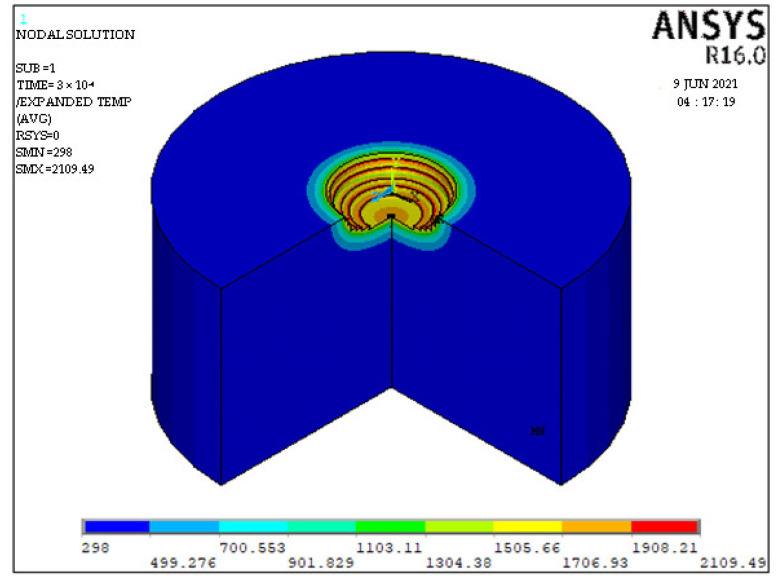
Predicted crater shape for Φ 40 μm at discharge duration 300 μs, peak current 6 A and gap voltage 80 V.

Figure 2 present the forecasted crater geometry using the FEM simulation model with 100% PFE for Φ40 μm powder mixed dielectric, respectively.

## 2. Materials and Methods

**Figure 3:**
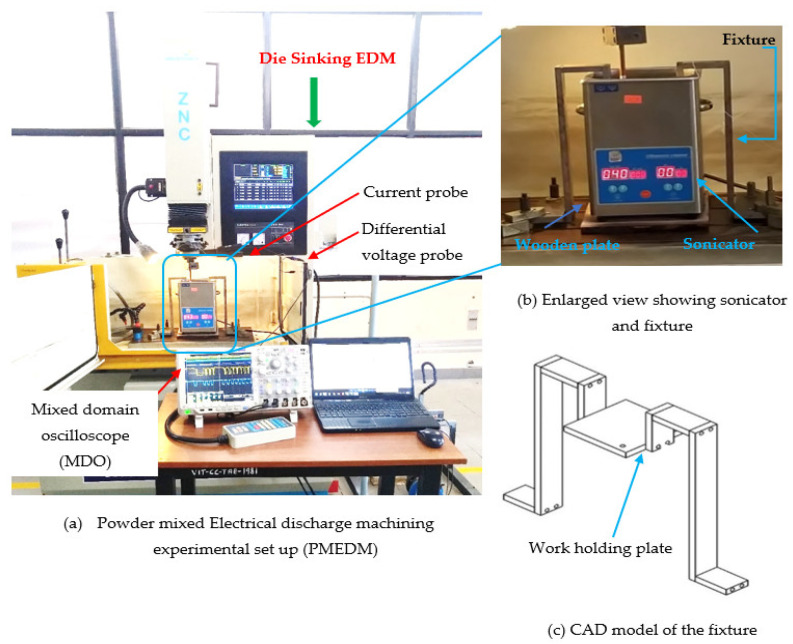
Experimental setup for PMEDM process.

The experiments are carried out using SMART-S 50 ZNC die-sinking machine (M/s. Electronica machine tools Ltd, Pune, India) equipped with a DC iso-pulse generator. The setup and the experimental conditions are presented in Figure 3 and Table 2, respectively. In this investigation an AISI 304 steel plate of dimension 100 × 100 × 5 mm^3^ is taken as the workpiece, and a pure copper rod of Φ 8mm is chosen as the tool electrode material. The chemical composition of the workpiece are C-0.07%, Cr-19.5%, Ni-10.5%, Mn-2%, Si-0.75%, P-0.045%, S-0.03% and the rest is Fe. The average thermo-physical properties of workpiece such as ρ-7910 kg/m^3^, c_p_-530 J/kg °C, T_m_-1454 °C and k-16.3 W/m °C, are considered for this study. Commercially available hydrocarbon oil (ELEKTRA supplied by M/s. Electronica machine tools Ltd, Pune, India) was chosen as the dielectric fluid. Molybdenum-di-sulphide powders of mean size Φ40 μm and Φ90 nm are chosen for this study. The discharge duration (T_d_), peak current (I_p_) and gap voltage (V_g_) are considered as input process parameters. Each input process parameter is chosen to cover wide range of operating conditions to meet industry requirements. During experimentation, all input parameters are varied using a one-factor-at-a-time (OFAT) approach.

To conduct PMEDM experiments on the die-sinking EDM machine, the existing dielectric circulation system was temporarily bypassed, and a separate experimental setup was developed using an ultrasonic vibrator with fixture attachments. Exposing the powder mixed dielectric to ultrasonic vibration promotes even dispersion; prevent agglomeration of powder particles and enhances the flushing conditions at the spark gap during machining. The ultrasonic vibrator (bath sonicator) is placed on the worktable and a wooden plate (for insulation) is kept between the bath sonicator and the machine’s worktable as shown in Figure 3b. The powder mixed dielectric liquid is exposed to a constant ultrasonic vibration frequency of 40 kHz. It is perceived from preliminary studies that, by exposing the powder mixed dielectric to continuous ultrasonic vibrations, the powder particles in the dielectric liquid start to agglomerate and settle at the bottom. Therefore, it is imperative to identify the maximum time duration for the powder particles to remain suspended within the dielectric liquid before agglomeration begins. The initial experimental studies revealed that, beyond 120 min of sonication, the powders start to sediment at the bottom. Therefore, the sonication time is kept constant at 120 min. For every 120 min, the fresh powder mixed dielectric is replenished. To hold the workpiece inside the ultrasonic vibrator, a new fixture is designed and the CAD model of the fixture is presented in Figure 3c. To record the pulses produced during machining, a four-channel digital mixed domain oscilloscope (MDO model: Tektronix MDO4104C) with 5 GSPS sampling rate is used. A differential voltage probe (Tektronix, HP9100, 100 MHz) and current probe (Model: Tektronix A622 AC/DC) were connected to MDO to capture voltage and current pulses, respectively. Each pulse train data is recorded for duration of 400 ms with 100,000 data points. The pulse frequency is assessed by adopting a thresholding approach [30] and a code is written in MATLAB R2021a (M/s. MathWorks, Natick, MA, USA) software to count the number of pulses. 

**Figure 4:**
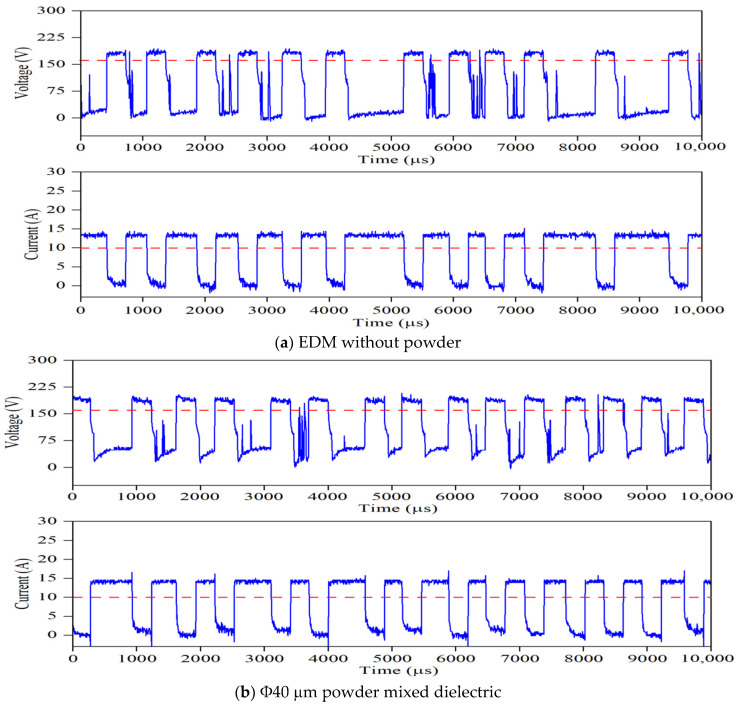

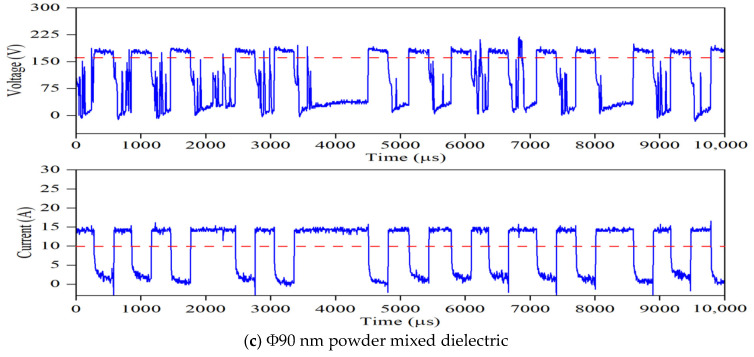
Typical voltage and current pulse train acquired for discharge duration 300 μs, peak current 6A and gap voltage 80 V (--- Threshold value).

**Figure 5:**
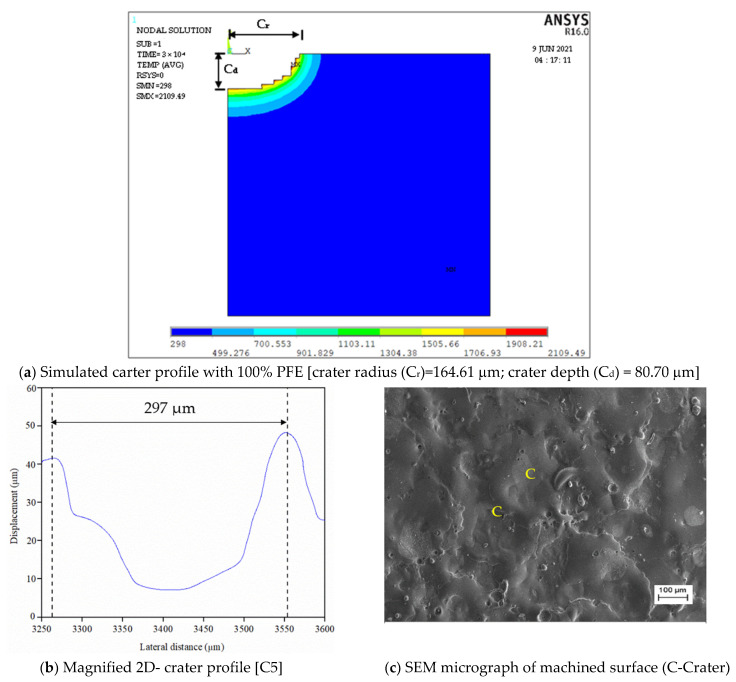

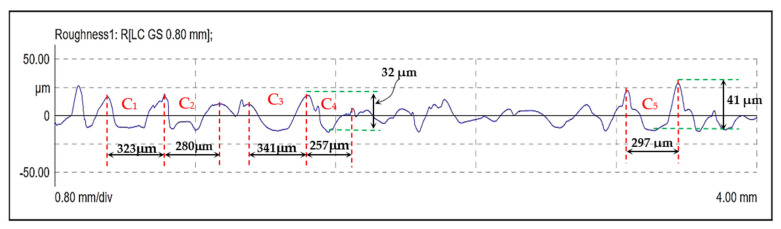
Simulated and experimentally formed carter geometry for Φ40 μm at T_d_ = 300 µs, I_p_ = 6 A, V_g_= 80 V.

**Figure 6:**
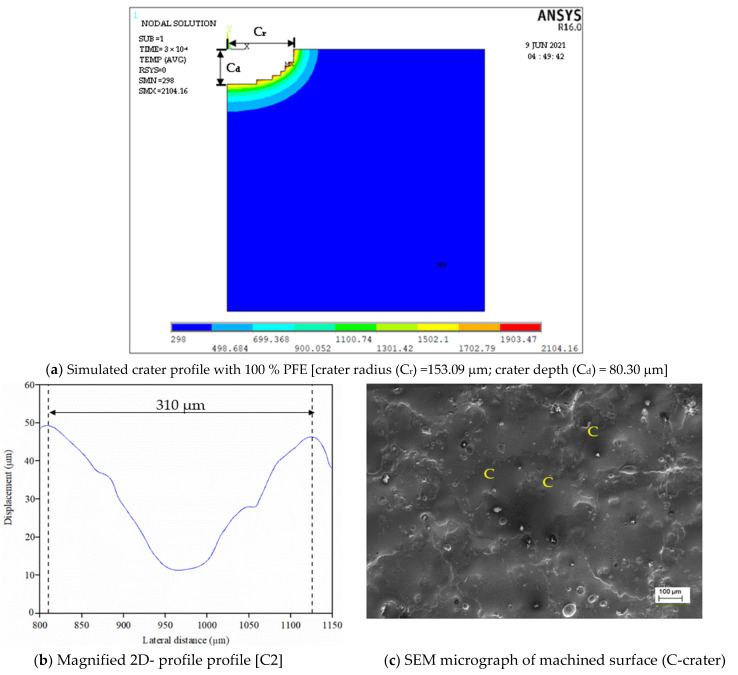

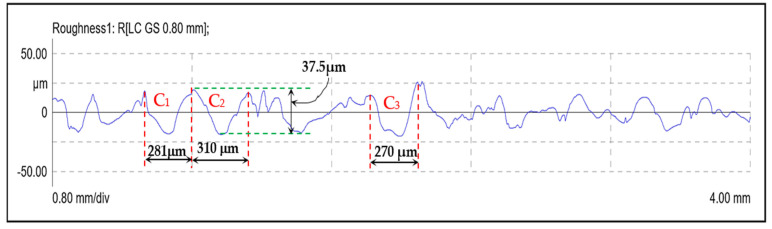
Simulated and experimentally formed carter geometry for Φ90 nm at T_d_ = 300 µs, I_p_ = 6 A, Vg= 80 V.

To assess the pulse frequency, first a set of experiments were conducted in ultrasonic vibration (40 kHz)-induced dielectric medium without powder for all input parameters. Further, experiments were extended by suspending each Φ40 μm and Φ90 nm MoS_2_ powder particles with concentration of 1 g/L of the dielectric liquid. Figure 4 shows a typical pulse train revealing the applied threshold approach for all three cases. For every input condition, pulse train data are captured at ten different intervals and its average value is taken for study, as presented in Table 3. The influence of the input process variables on crater geometry, such as crater diameter and crater depth, is analysed. The crater diameter is assessed using scanning electron microscopy (make ZEISS EVO 18 SEM, M/s. ZEISS International, Oberkochen, Germany) images. The crater diameter is measured at five different locations on the machined surface and its average value is considered for comparison. By knowing the experimentally formed crater diameter, the depth of crater can be assessed from the 2D roughness profile [24]. The surface roughness of the machined sample is measured using “MarSurf GD120 (M/s. Mahr GmbH, Goettingen, Germany) roughness tester at five different locations. In each roughness profile, the crater diameter is identified and its corresponding depth values are taken. A typical FEM simulation showing the crater geometry, SEM micrograph with crater diameter and 2D roughness profile with crater depth are presented in Figure 5 and Figure 6. The average of five crater depths identified from the 2D roughness profile is taken for assessment. To validate the proposed model, experiments were conducted by varying the process parameters encompassing finish, semi-finish, and rough machining conditions employed in the industry. During experimentation, constant ultrasonic vibration is imparted into the dielectric medium using the bath sonicator to promote enhanced flushing of the spark gap.

**Figure 7:**
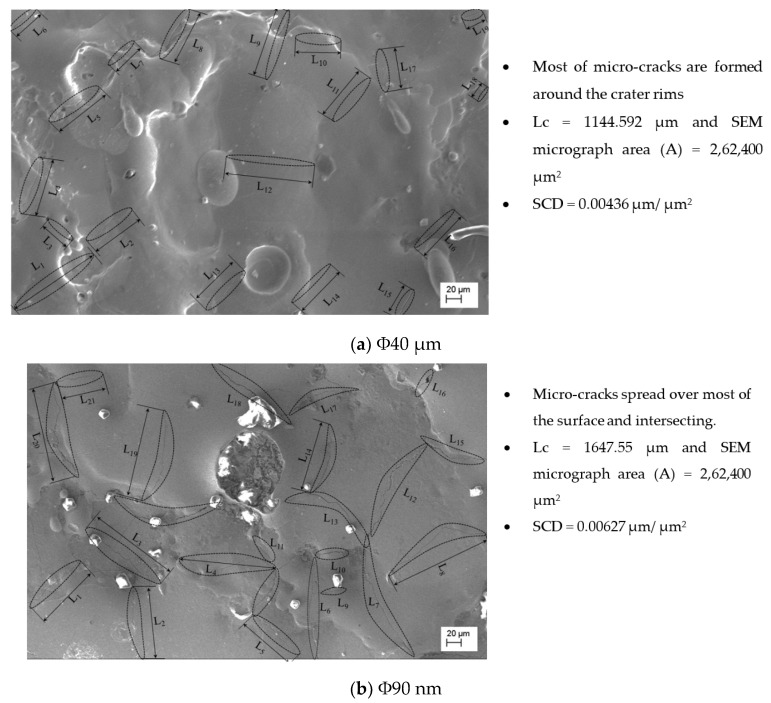
SEM micrographs at 500X for SCD at T_d_ = 300 µs, I_p_ = 6 A, V_g_ = 80 V.

The study on surface crack density (SCD) is carried using SEM micrographs taken at 500× magnification. The SCD is the ratio of total crack length to area of SEM micrograph and is evaluated using Equations (7) and (8).

(7) $$L_{c}=L_{1}+L_{2}+L_{3}+L_{4}+L_{5}\ldots \ldots L_{n}$$

(8) $$\mathrm{SCD}=\frac{L_{c}}{A}$$

where, L_c_ is the total length of micro-cracks (µm), L_1_, L_2_...L_n_ the length of each micro-crack (µm), A is measured area (µm^2^) of the SEM micrograph. Typical SEM micrographs of machined surfaces with Φ40 μm and Φ90 nm powder and measurement procedures are presented in the Figure 7. The skewness and kurtosis values are considered from the measured surface roughness data. The experimental conditions are stated in Table 2.

## 3. Results and Discussions

The effect of each input process variable and powder particle size on pulse frequency is presented in Table 3. Using the OFAT approach, the input process variables are varied. It is noticed from Table 3, the pulse frequency keeps reducing as the magnitudes of discharge duration, peak current and gap voltage rise. On the contrary, the pulse frequency grows with higher magnitudes of duty factor. For higher values of duty factor, a greater number of discharges occur in a given time (ELECTRONICA technology manual) that resulted in an increased number of pulses. In addition, the number of pulses occurred in PMEDM is slightly more than that produced in EDM without powder. As seen in Table 3, except a few settings, for almost all experimental conditions, the K_n_ varies from 1 to 1.67. The effect of input parameters on crater diameter and crater depth is analysed by varying one input parameter, whereas other parameters are maintained constant. The influence of individual input parameter on crater geometry and chemical alteration of machined surface produced by Φ40 μm powder mixed dielectric (hereafter mentioned as ‘Φ40 μm’) and Φ90 nm powder mixed dielectric (hereafter mentioned as ‘Φ90 nm’) is attempted. To compare the simulated results with experimentally data, a PFE of 95% and 45% is used for crater diameter and crater depths respectively are presented in Table 4.

### 3.1. Discussion on the Influence of Powder Suspended into Dielectric Liquid

**Figure 8:**
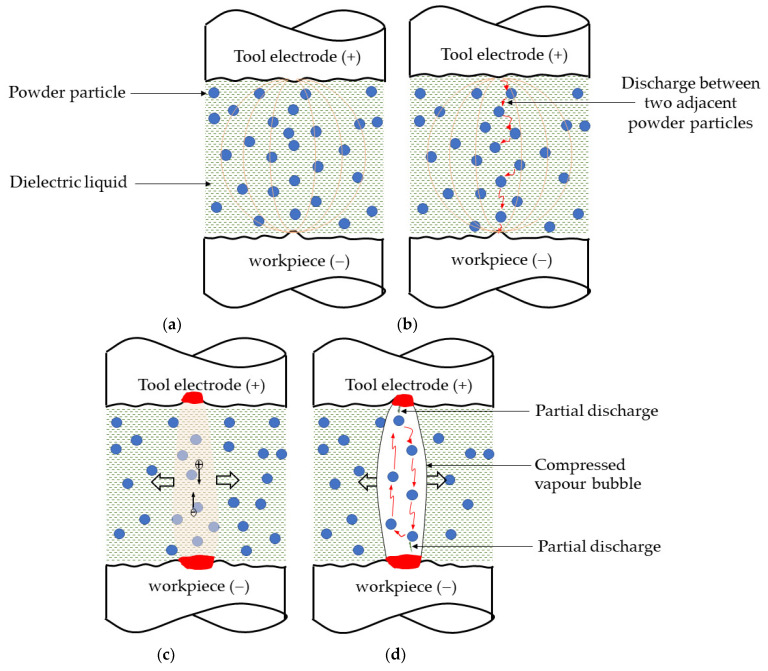
Schematic representations for partial discharge phenomena in PMEDM process. (**a**) Electric field aberration (**b**) Series discharge occur in-between the powder particles (**c**) Dis-charge channel formation (**d**) Occurrence of partial discharges.

The suspension of conductive powder particles into dielectric liquid alters the discharge characteristics of EDM process as explained using Figure 8. In PMEDM process, a high potential difference is applied between the electrodes separated by spark gap filled with powder suspended dielectric. The presence of stray ions and conductive powders in the gap aids to intense electric field aberration at the region of least electrical resistance between the electrodes as represented in Figure 8a. Numerous positive and negative ions gather around the periphery of the powder particle [50]. The powder particles lying in the least electrically resistant region align themselves to form a chain [51]. Each conductive particle acquires electric charge and is expressed by Equation (9) [48,49,53] as

(9) $$Q_{p}=\frac{2}{3}{\pi \varepsilon }_{0}\varepsilon_{r}R^{2}E_{0}$$

where ε_0_ is the permittivity of free space, ε_r_ is the relative permittivity of fluid, R is the radius of particle, E_0_ is applied filed (V/d). For an applied potential difference between the electrodes (tool and workpiece), each individual powder particle acts as a charge carrier and the magnitude of charge each particle holds is dependent on its size (radius (R)). As and when the electrical density between the two adjacent particles exceeds the dielectric strength of liquid, electrical breakdown (discharge) occurs between these two powders [50]. This discharge leads to the short circuit of two powders followed by redistribution of charges. Furthermore, charges accumulate on the periphery and the next discharge happens between these two powders and the adjacent powder particle. Subsequently a series of discharges occur within other powders as shown in Figure 8b; finally, an early discharge takes place between the electrodes at the place with the least electrical resistance. A discharge channel formed between the tool and workpiece around the powder particles as shown in Figure 8c. As the discharge remains, the continuous melting and vaporization of dielectric liquid, powder particles and electrodes results in the formation of a compressed vapour bubble or plasma. Now that the discharge channel is perfectly conducting [48] upon the applied electric field, the powder particles acquire an electric force owing to continuous bombardment of electrons. When the electric force exceeds the force of gravity, the particles accelerate towards the oppositely charged electrode. Particle behaviours are influenced by the medium’s (compressed vapour bubble) properties and the charge exchange between the medium and the particle. As the particles approach an electrode, the induced electric field produces a partial discharge before it strikes an electrode. Immediately after the partial discharge, the magnitude and orientation of induced electric force on the particle changes. However, the particle keeps on moving until it loses its inertia without making physical contact with the electrode [49] as explained in Figure 8d.

### 3.2. The Effect of Discharge Duration on Crater Geometry

**Figure 9:**
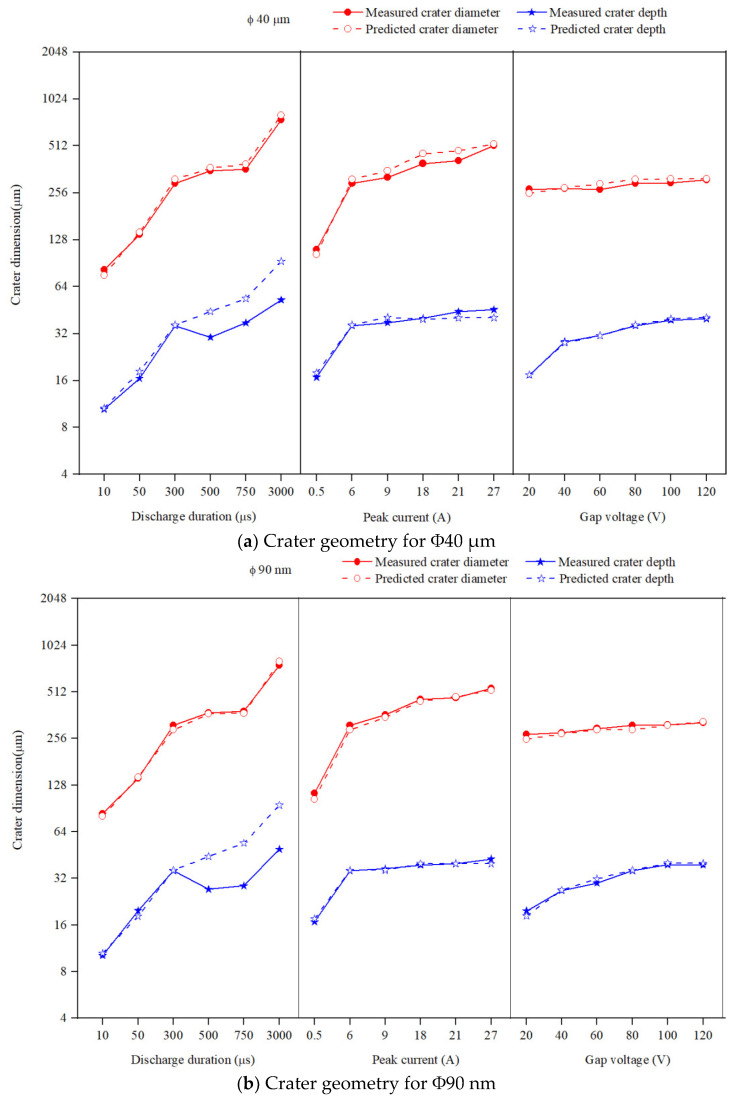
Effect of input process variables on crater geometry.

The effect of discharge duration on crater geometry is analysed by varying the discharge duration from 10 to 3000 μs keeping peak current, and gap voltage at 6 A, and 80 V respectively. Figure 9 depicts the effect of discharge duration on crater diameter in both Φ40 μm and Φ90 nm. The carter diameter grows with increases in discharge duration magnitudes. As the magnitude of discharge duration rises, the diameter of compressed vapour bubble raises, which resulted in increased crater diameter. The crater diameter generated by Φ90 nm is slightly larger than that produced by Φ40 μm. As mentioned in Equation (9), the charge carried by a particle grows with particle size (R), which means the charge carried by each Φ40 μm is larger than Φ90 nm particle. Yet, for a given powder concentration suspended inside the dielectric liquid, the Φ90 nm contains more powder particles than Φ40 μm. With Φ90 nm, the increase in the number of particles creates more charge carriers which subsequently acquire more energy that results in a larger compressed vapour bubble. The increase in size of the compressed vapour bubble produces a greater crater diameter for Φ90 nm than is produced by Φ40 μm. As the size of the compressed vapour bubble raises, the energy density inside the bubble drops, which results in the formation of a shallow crater [50]. In comparison with the experimental data, the simulated results predict a crater diameter with an average absolute error of 3.34% and 0.19% for Φ40 μm and Φ90 nm respectively. The influence of discharge duration on crater depth is illustrated in Figure 9. As is shown in Table 4, up to the discharge duration of 300 µs, the experimentally formed crater depth is in close agreement with the simulated results with an average absolute error of 4.43% and 4.09% for Φ40 μm and Φ90 nm, respectively. However, for larger values (of discharge duration >300 μs), the simulation results over predicts in comparison with the experimentally measured crater depth for both Φ40 μm and Φ90 nm. At larger magnitudes of the discharge duration, as the discharge begins, the energy intensity inside the compressed vapour bubble is large.

As the discharge remains, owing to the continuous melting and evaporation by electro-thermal heating, the size of the compressed vapour bubble starts to grow radially [27,28]. The energy produced due to electro-thermal heating starts to spread over the workpiece surface inside the vapour bubble. The heat is spread over the periphery rather than conducted into the workpiece. Subsequently the energy density inside the vapour bubble drops [50]. At the end of discharge, owing to the lowered energy intensity inside the vapour bubble, the material is melted only at the periphery of the spark vicinity that resulted in smaller crater depth. For all discharge durations, the crater depth produced by Φ90 nm is smaller than that produced by Φ40 μm, which can be seen in Figure 9.

**Figure 10:**
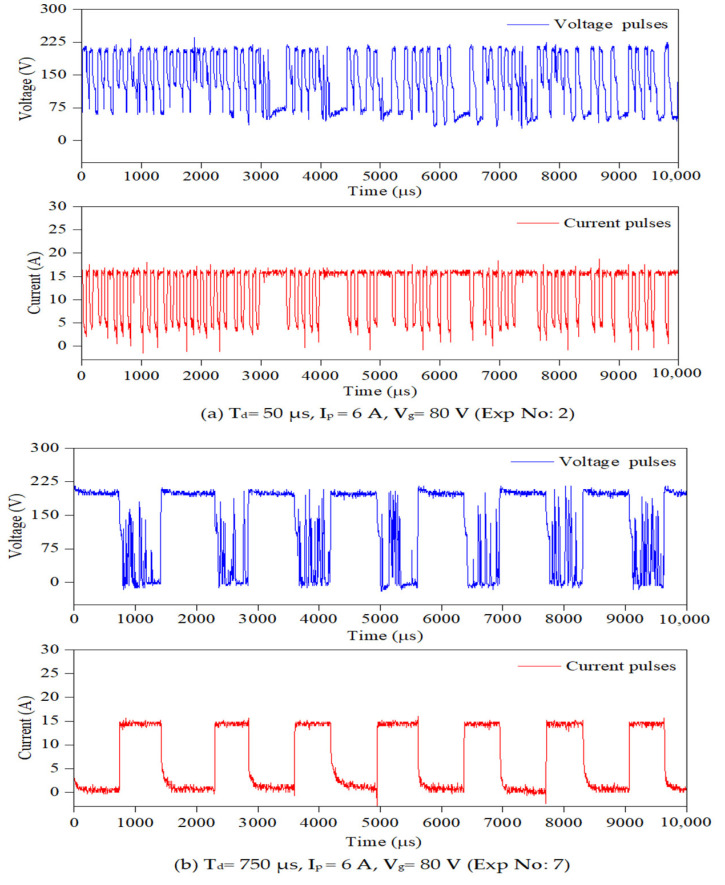

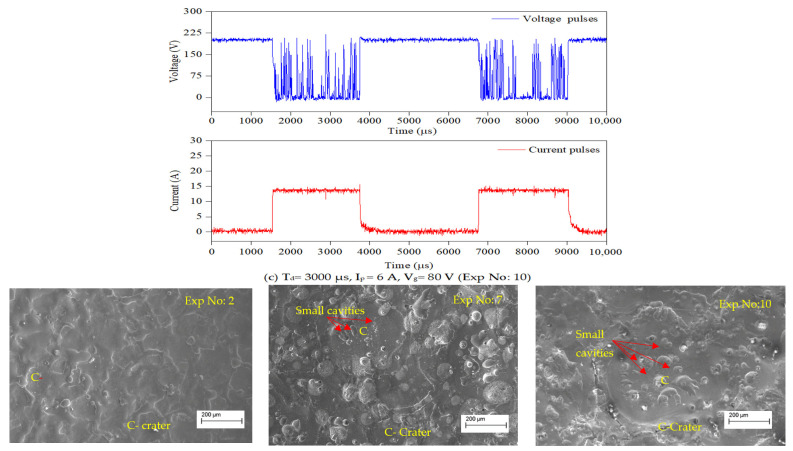
Typical pulse trains data and SEM micrographs for Φ40 μm at various discharge durations (C-crater, PD-partial discharge).

**Figure 11:**
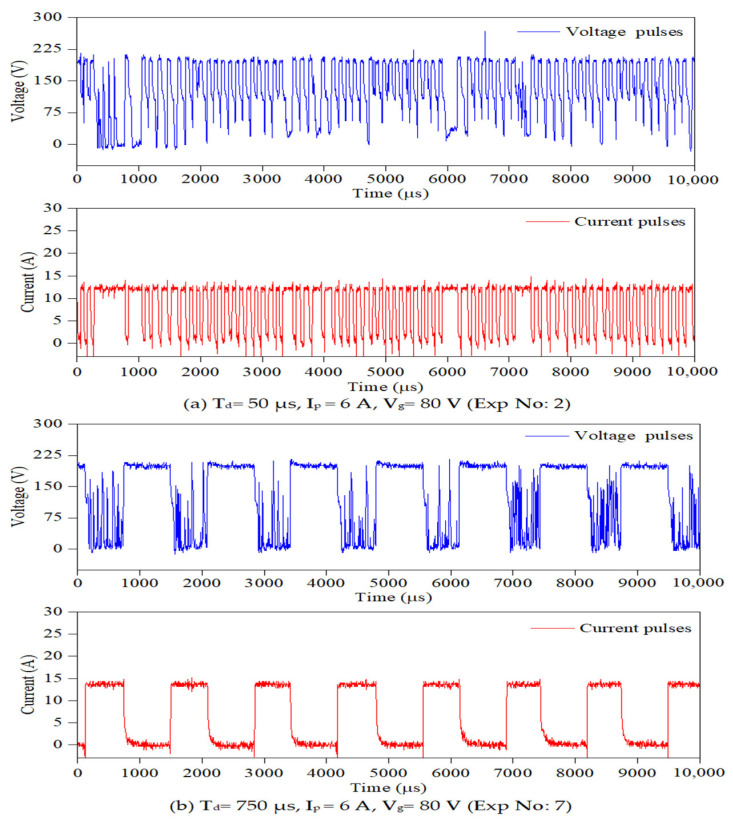

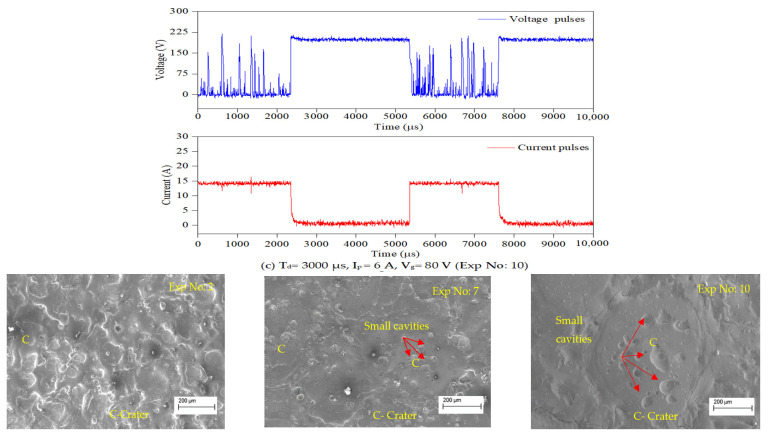
Typical pulse trains data and SEM micrographs for Φ90 nm at various discharge durations (C-crater, PD-partial discharge).

Further investigation is carried out on the crater morphology using SEM analysis. The SEM micrograph of a machined surface produced by Φ40 μm and Φ90 nm for varying discharge durations are presented in Figure 10 and Figure 11 respectively. The SEM micrograph revealed that, small cavities are formed within the primary crater at higher discharge durations (>300 μs) for both Φ40 μm and Φ90 nm. These small cavities are formed due to the occurrence of partial discharge (PD). However, at smaller discharge durations (up to 300 μs) cavities within the crater is not identified. By increasing the magnitudes of discharge duration, the charge carriers (powder particles) within the discharge channel gain more energy, move towards the workpiece and produce PD within the primary crater. The number of PD grows for longer discharge durations. The energy transferred by these PD is minimal, as it only could melt a minute portion of material. In addition to these observations, an attempt has been made to identify these PD phenomena using the voltage and current pulse train captured during experimentation. Through pulse trains, the occurrence of PD within the primary discharge is recognised clearly in both Φ40 μm and Φ90 nm. In both Figure 10 and Figure 11, the PD is clearly distinguished from the voltage pulse train and fast raising current pulses [53] detected at longer discharge durations (Figure 10b,c and Figure 11b,c).

### 3.3. The Effect of Peak Current on the Crater Geometry

In this work, the peak current is varied from 0.5 to 27 A keeping other parameters constant at 300 μs, and 80 V for discharge duration, and gap voltage respectively. In PMEDM, each electrical discharge produces a discharge spot by erosion of material from the work surface. As a result, a crater is formed and the size of the crater depends on the energy transferred into the workpiece material. The energy intensity grows with raise in peak current magnitudes thus leading to an increased erosion volume [2,17]. As a result, large sized craters form on the machined surface. From Table 4, it is noticed, the Φ90 nm produced bigger crater diameter compared to Φ40 μm for all current magnitudes. When compared with the experimentally formed crater diameter, the developed model for Φ90 nm forecasts crater diameter with an average absolute error of 3.65% and 7.32% for Φ40 μm.

**Figure 12:**
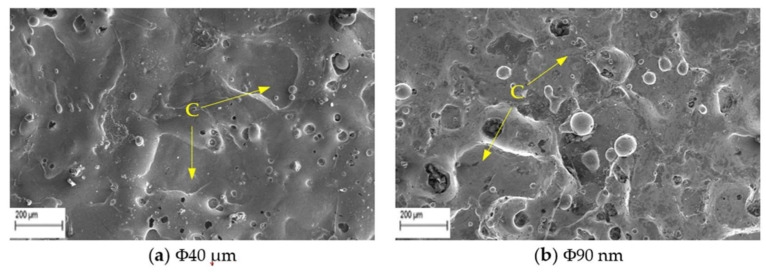
SEM micrograph of surface machined using T_d_ = 300 µs, I_p_ = 18 A, V_g_ = 80 V (C-crater).

The impact of peak current on experimentally formed crater depth is presented in Table 4. As can be seen in Table 4, the increase in peak current magnitudes produces a deeper crater on the machined surface in Φ40 μm than Φ90 nm. For a known peak current value, the energy transfers into the workpiece surface due to electro-thermal heating by conduction. As the peak current magnitude rises, more eroded material forms on the machined surface that results in deeper craters forming, which is evidenced from the experimental data listed in Table 4. The results reveal that, the Φ40 μm yields deeper crater than that created by Φ90 nm. In comparison with experimentally formed crater depth, the simulated results of Φ40 μm and Φ90 nm estimate crater depth with an error of 1.06% and 0.23%, respectively. The investigation of SEM micrograph for varying current magnitudes revealed that partial discharges are not prominent for varying current magnitudes. Figure 12 shows the SEM micrograph of Φ40 μm and Φ90 nm revealing the absence of PD.

### 3.4. The Effect of Gap Voltage on Crater Geometry

The gap voltage is varied from 20 to 140 V and the other parameters discharge duration, and peak current, are maintained constant at 300 μs, and 6 A respectively. Figure 9 shows that the crater diameter grows with raise in gap voltage magnitudes. At a lower gap voltage, the electric field intensity is reduced, which results in small carters forming on the workpiece. As the gap voltage value rises, the increase in the electric field intensity at the discharge gap ends in forming large carter diameter. The developed model predicts crater diameter with an error of 2.76% and 2.78% for Φ40 μm and Φ90 nm respectively.

**Figure 13:**
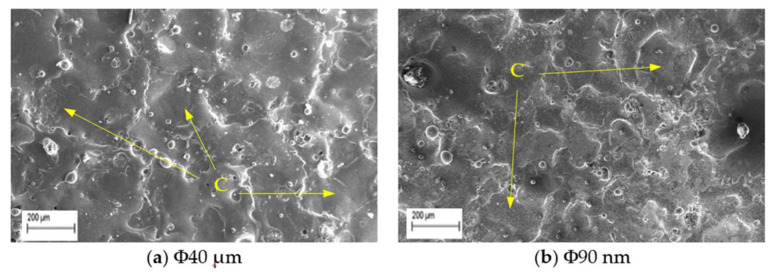
SEM micrograph of surface machined using T_d_= 300 µs, I_p_ = 6 A, V_g_= 100 V (C-crater).

The experimentally formed crater-depth magnitude rises with increase in gap voltage values. By increasing the gap voltage intensity, owed to the enlargement of the discharge gap, the energy transferred into the workpiece reduces and results in the formation of shallower craters than that formed with discharge duration and peak current. Table 4 reveals that, the Φ40 μm produces deeper crater whereas Φ90 nm produces shallower craters. The projected model predicts crater depth with an error of 0.52% and 0.80% for Φ40 μm and Φ90 nm respectively for varying gap voltage magnitudes. Figure 13 shows the SEM micrograph of Φ40 μm and Φ90 nm machined surface showing the nonexistence of PD.

### 3.5. Analysis of Surface Crack Density

**Figure 14:**
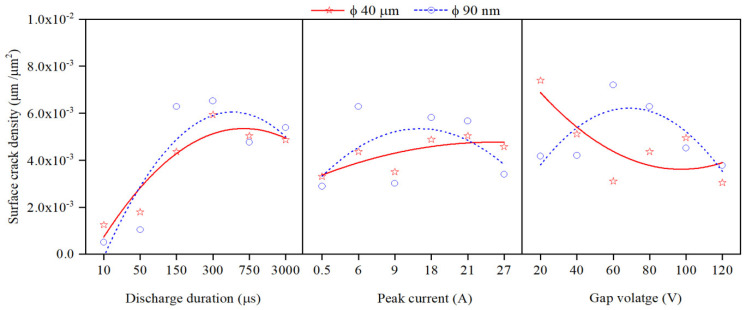
Effect of input process parameters on surface crack density.

**Figure 15:**
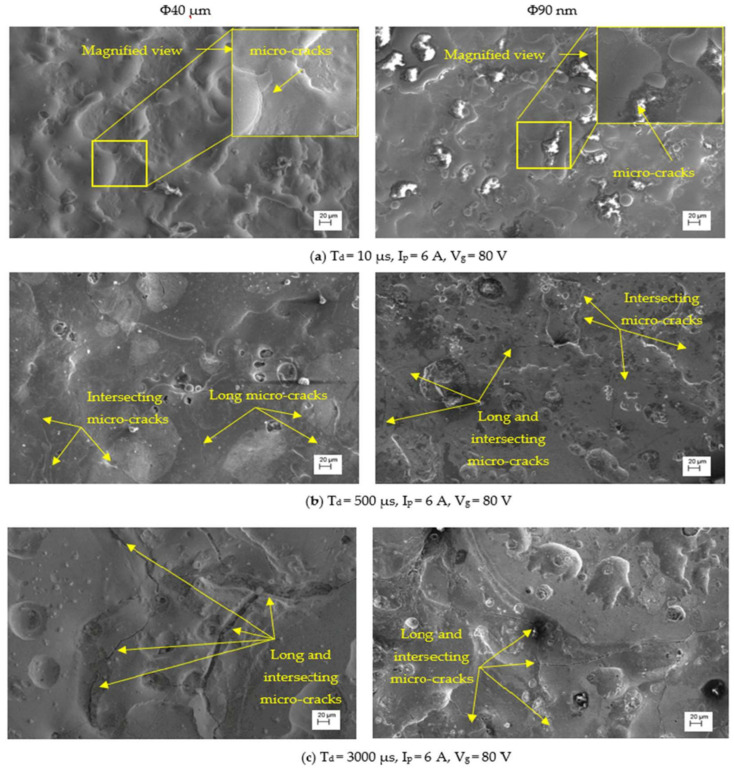
SEM micrographs with various discharge durations.

**Figure 16:**
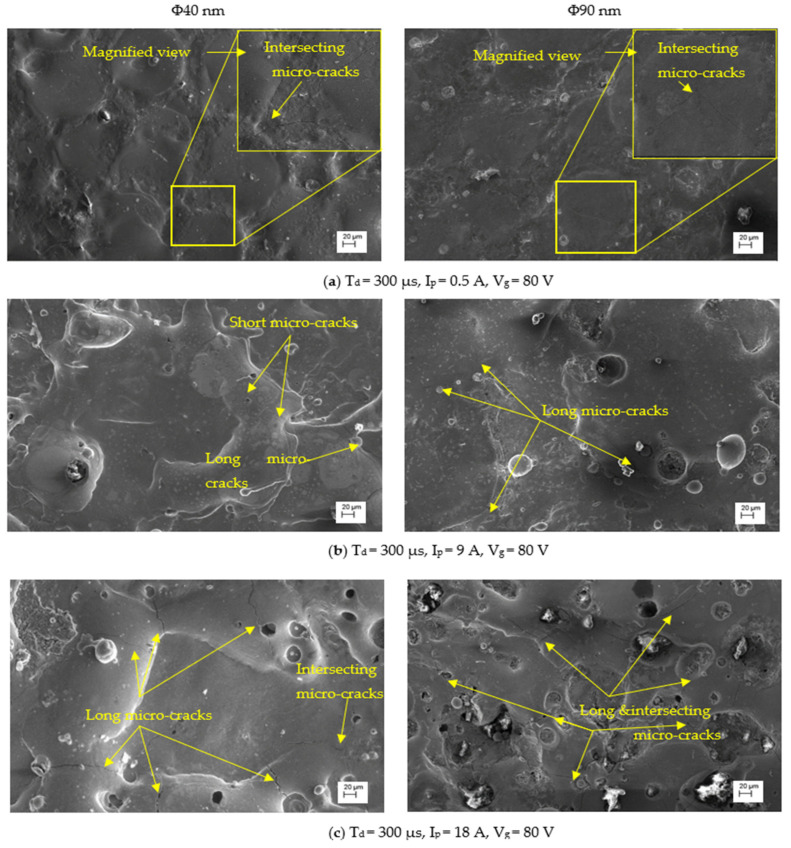
SEM micrographs with variations in peak current.

The SCD of machined surfaces is assessed using Equation (8) and the significance of input parameters on SCD of machined surface is presented in Figure 14. The SEM micrographs of the PMEDMed surface of AISI 304 steel specimen under varying discharge duration are presented in Figure 15a–c. It is evident from SEM micrograph that the discharge duration has direct influence on the micro-cracks. The investigation discloses that the density of surface cracks varied non-linearly with discharge duration [38]. From Figure 14, it is perceived that the Φ90 nm produces lessened micro-cracks for low magnitudes of discharge duration (upto 50 µs) compared to Φ40 μm. In Φ90 nm, at discharge duration of 10 µs and 50 µs, the SCD decreased significantly by 58.97% and 41.72% respectively compared to Φ40 μm. From Figure 15, it is apparent that the increase in discharge duration produces severe micro-cracks. This is due to successive electrical discharges, intense heat, local melting and vaporization of work material causes raise in thermal gradient that resulted in augmented residual stress [39]. As a result, the surface crack density increases. The trend line demonstrates that, the SCD grows until 300 µs discharge duration in both Φ40 μm and Φ90 nm. Further raising the discharge duration magnitudes, the SCD starts to diminish in both Φ40 μm and Φ90 nm. At longer discharge durations, the reduction in the energy intensity within the plasma melt lesser amounts of work material. Subsequently, lessened micro cracks form on the machined surface. Figure 16 illustrates the SEM micrographs showing the micro-crack of specimen machined under various peak current magnitudes. At a peak current magnitude of 0.5 A, long, narrow and intersecting micro-cracks are formed on the machined surface in both Φ40 μm and Φ90 nm, as seen in Figure 16a. The SCD of the Φ90 nm is lowered by 12.45% compared to Φ40 μm. By raising the peak current magnitudes, SCD varies nonlinearly. 

**Figure 17:**
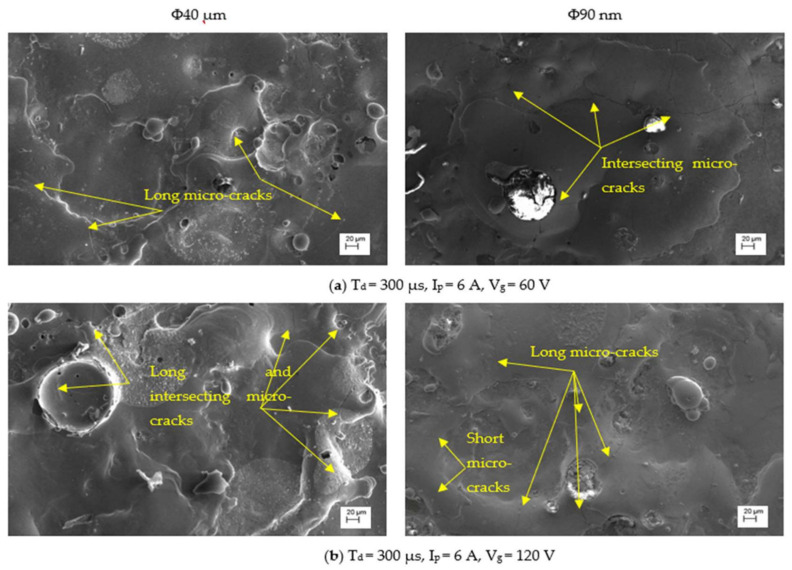
SEM micrographs with variation of gap voltage.

The trend line shows that the SCD grows up to a peak current magnitude of 6 A; thereafter, SCD starts to decline. The micro-cracks were observed around the crater and on the crater rims. In comparison with 0.5 A, the micro cracks forming at 6 A are short and intersecting each other for both Φ40 μm and Φ90 nm. After a further increase in the peak current magnitude up to 27 A, the Φ90 nm produces 25.81% lessened SCD compared to that of Φ40 μm. Longer and wider penetrating micro-cracks are formed at higher magnitudes of the peak current [19]. Figure 17 depicts the significance of gap voltage on SCD in both Φ40 μm and Φ90 nm. At lower gap voltage setting, the spark gap is narrow, the intense electric sparks impinge the worksurface with high-impact force which causes more material to melt and re-solidify. This results in the formation of more micro-cracks. At 20 V gap voltage, the Φ90 nm produce 43.58% lessened micro-cracks compared to Φ40 μm. Further increasing the gap voltage, due to a widened spark gap between tool and workpiece, less energy transferred into the work surface; thus, less material melted, and as a result, less SCD formed on the machined surface [37,39] in both Φ40 μm and Φ90 nm. Among all parametric conditions at the gap voltage 60 V, the minimum SCD of 0.003 µm/µm^2^ is achieved in Φ40 μm.

### 3.6. Analysis of Skewness (R_sk_) and Kurtosis (R_ku_)

**Figure 18:**
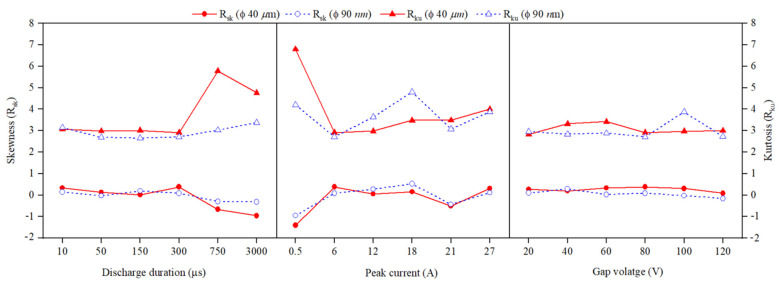
Effect of input process parameters on Skewness and Kurtosis.

The skewness (R_sk_) and kurtosis (R_ku_) can be used to assess the micro-geometrical characteristics of machined surfaces. The skewness is a measurement of the surface profile’s asymmetry relative to its mean line. Negative R_sk_ suggest a surface with a predominance of sharp valleys and rounded peaks, whereas positive R_sk_ shows a surface with predominance of rounded valleys and sharp peaks. The R_ku_ of the surface profile is a measure of the degree of peaking. If R_ku_ > 3, the surface profiles have many high peaks and valleys; consequently, R_ku_ < 3, reveals surface profiles with low peaks and valleys [39,41]. A typical skewness–kurtosis envelope shows that, the EDM process is capable of producing surfaces with positive skewness and kurtosis around 3 [40,54]. From the experimental results (Figure 18) it is perceived that, using MoS_2_ powder-suspended dielectric, surfaces with negative skewness is easily obtainable at T_d_ > 750 μs and I_p_ = 0.5 A conditions for both Φ40 μm and Φ90 nm. Compared to Φ40 μm, the surfaces with negative skewness are easily obtained using Φ90 nm for higher magnitudes of discharge duration and gap voltage. A minimum skewness of −1.4 is achieved at peak current of 0.5 A for Φ40 μm. Surfaces with negative skewness offer good bearing capacity and lubricant retention capacity [40]. By varying the discharge duration between 50–300 μs and gap voltage between 40–80 V, the kurtosis of the machined surface less than 3 is achieved. These machined surfaces offer good gripping and locking ability [39,40,41]. These results indicate that using PMEDM process tailor made surface texture can be produced on the machined surface by selecting appropriate discharge parameter combinations.

### 3.7. Effect of Process Parameters on Chemical Alteration of Machined Surface

**Figure 19:**
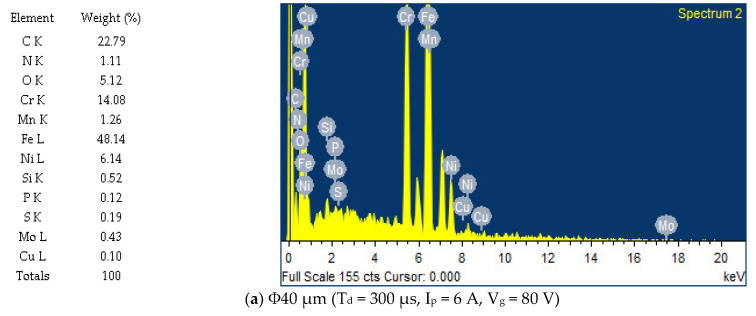

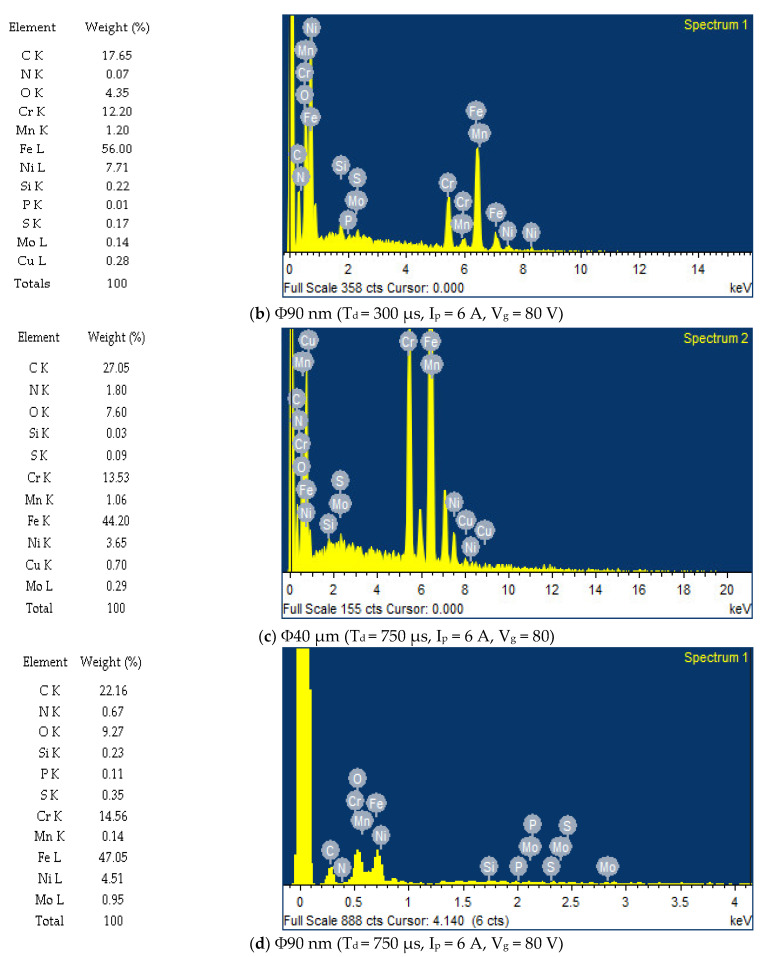

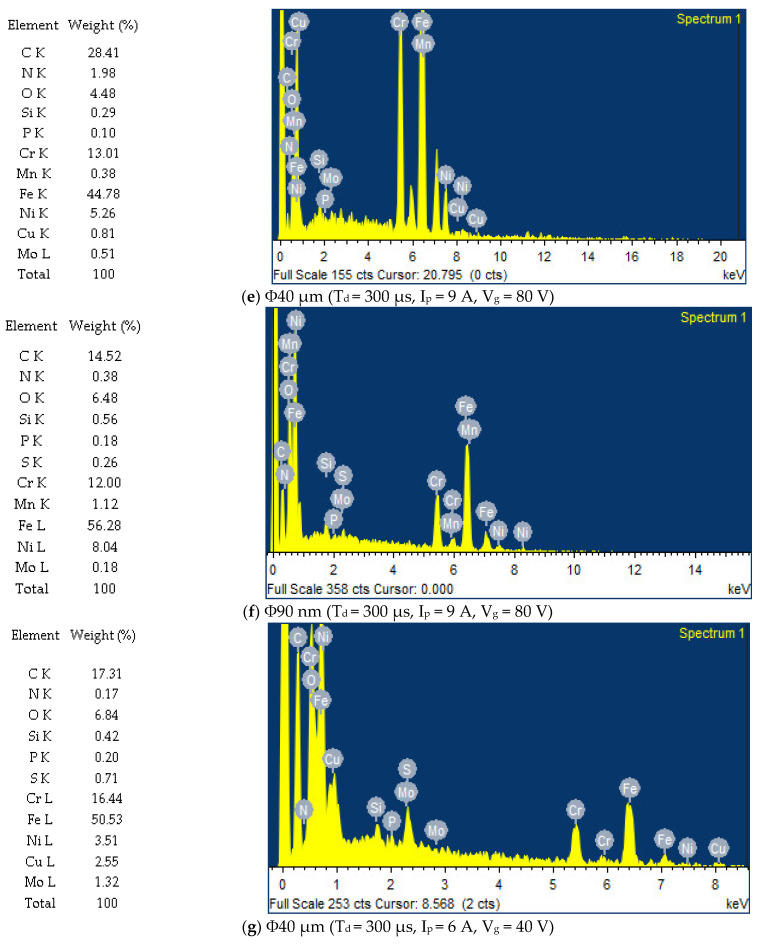

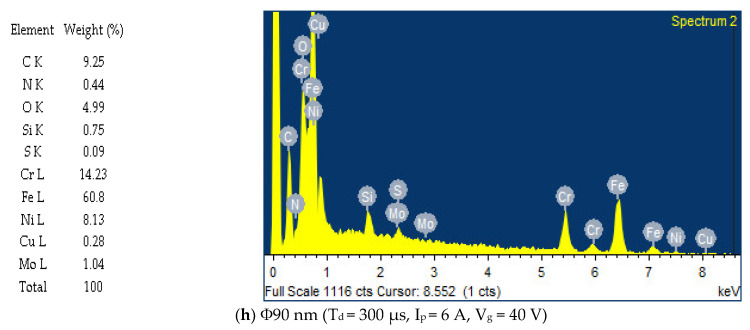
EDS analysis under various input parameters.

**Figure 20:**
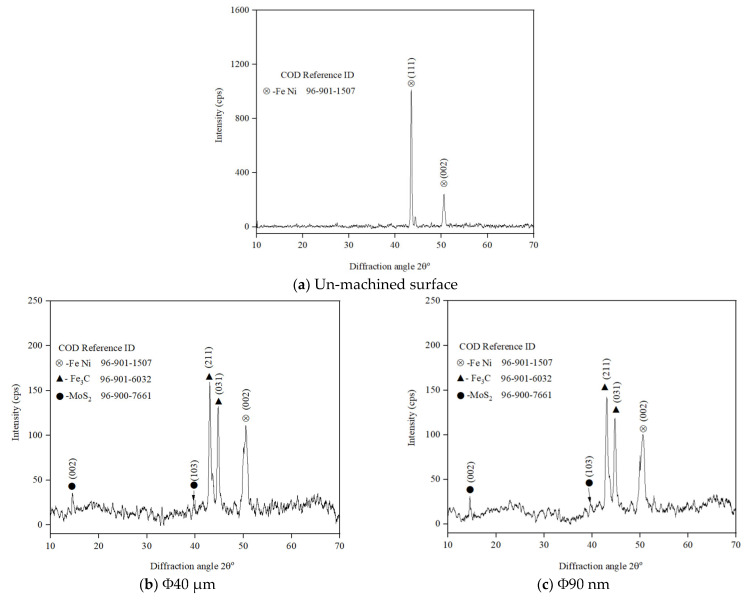
XRD pattern of workpiece surface.

The effects of discharge duration, peak current, and gap voltage on the chemical alteration of the machined surface are analysed and presented in Figure 19. It is perceived from Figure 19, for both Φ40 μm and Φ90 nm, that by increasing the discharge duration (Figure 19a–d) peak current (Figure 19a,b,e,f), gap voltage (Figure 19a,b,g,h) magnitudes, more carbon, oxygen and copper form on the machined surface owed to pyrolysis effect. It is to be noticed from the energy dispersive spectroscopic profiles that the MoS_2_ powder particle is deposited on the machined surface on components machined under a lower gap voltage of 40 V (Figure 19g,h) and peak current 9 A (Figure 19e,f). For all operating conditions, more MoS_2_ powder particles were deposited on surfaces machined using Φ40 μm, as observed from EDS profiles. Further investigation is carried out to confirm the deposition of MoS_2_ particles through X-ray diffraction analysis. Figure 20a shows the XRD profiles of un-machined surface and the surface machined at T_d_ = 300 µs, I_p_ = 6 A and V_g_ = 100 V for Φ40 μm and Φ90 nm are presented in Figure 20b and Figure 20c, respectively. The XRD profiles reveal the presence Fe_3_C in lattice planes (211) and (031). In addition, the deposition of MoS_2_ powder particles is identified at (002) and (103), corresponding to lattice planes on both Φ40 μm and Φ90 nm machined surfaces.

## 4. Conclusions

In this study, an axisymmetric two-dimensional thermal model is developed to predict the crater morphology of AISI 304 steel surfaces formed by suspending MoS_2_ powder particles into the dielectric in the EDM process. Experiments were conducted using MoS_2_ powder particles of mean size Φ40 μm, Φ90 nm and by selecting a wide range of input process parameters. Using the OFAT approach, the impact of discharge duration, peak current and gap voltage on crater morphology and chemical alteration in machined surfaces are analysed. During experimentation, one parameter is varied at a time by keeping the remaining input parameters constant; a summary of the proposed work is listed below.

- With the rise in discharge duration magnitudes, the crater diameter and depth grow proportionately for both Φ40 μm and Φ90 nm. However, the Φ90 nm produces a large crater diameter, whereas the Φ40 μm produces a deeper crater on the machined surface. In comparison with the experimentally formed crater geometry, the proposed model predicts crater diameter with an average absolute error of 3.34% and 0.19% for Φ40 μm and Φ90 nm, respectively. Consequently, for crater depth, the proposed model holds good up to 300 μs discharge duration. At larger magnitudes of discharge duration (>300 μs), owing to the lessened energy intensity inside the compressed vapour bubble, the melted material is not completely expelled from the spark vicinity, which leads to a reduced crater depth. This resulted in over-prediction compared to the experimental data. A partial discharge phenomenon is noticed at higher values of discharge duration. Using SEM micrographs and pulse train data, the partial discharge is conceived.
- For each peak current magnitude, the Φ90 nm powder produces large diameter crater, consequently the Φ40 μm powder produce craters with greater depths. With the rise in peak current values, the energy intensity inside the vapour bubble grows, resulting in the formation of larger and deeper craters. In comparison with the experimental results, the developed model forecasts crater diameter with 7.32% and 3.65% error for Φ40 μm and Φ90 nm respectively. The simulated result predicts crater depth with an error of 1.06% and 0.23% for Φ40 μm and Φ90 nm respectively.
- Varying the gap voltage from small to large magnitudes, crater dimension grows proportionately for both Φ40 μm and Φ90 nm powder. The developed model estimates crater diameter with an error of 2.76% and 2.78% for Φ40 μm and Φ90 nm respectively. In comparison to the experimentally formed crater depth, the simulated model estimates with an error of 0.52% for Φ40 μm powder and 0.80% for Φ90 nm powder.
- Compared to Φ40 μm, the Φ90 nm condition produced surfaces with lessened SCD for discharge duration 10 μs, 50 μs; peak current 0.5 A and gap voltage 20 V. The raise in input parameter magnitudes, more severe micro-cracks are noticed on machined surface.
- In both Φ40 μm and Φ90 nm, the negative skewness is identified on surfaces machined with discharge duration 50 μs, 750 μs, 3000 μs; peak current 0.5 A and gap voltage 80 V, 100 V, 120 V.
- The chemical alteration of the machined surfaces is analysed for both Φ40 μm and Φ90 nm by varying input parameters. As is perceived from the EDS profile, more MoS_2_ powder deposits on surface machined with Φ40 μm than Φ90 nm. The XRD profile revealed the presence of MoS_2_ at (002)] and (103) lattice planes on both Φ40 μm and Φ90 nm machined surfaces.

The proposed work can be extended for the deposition of varied powder particles on difficult-to-machine material surfaces, producing components with improved load-bearing capacity using the powder mixed electrical discharge machining process.

## Figures and Tables

**Table 1:** Comparison on existing literature and contribution of proposed work in PMEDM process.

Existing Literature	Novelty of the Proposed Work
Powders used	
Micron sized powder (<60 μm) particles like Gr, Si, SiC, Ti, Cu, Al, Fe, MoS_2_; nano sized powders like Ag, CNT, MWCNT were suspended into the dielectric and reported their performance [5,7,8,9,10,11,30,31,37,42,43,44,45,46,47,48,49,50,51]	The combined effect ultrasonic vibration and powder particle size on the performance of PMEDM process is not yet explored. In this study, the impact of micron-sized (Φ40 μm) and nano-sized (Φ90 nm) MoS_2_ powder suspended dielectric on surface morphology, namely crater diameter, crater depth, surfaces crack density, skewness, kurtosis and chemical alteration of machined surfaces, is explored.
Modelling of PMEDM Process	
FEM modelling of the PMEDM process is reported using a constant pulse frequency factor [5,15,16]; ANOVA [42]; Box–Behnken design [43]; Grey relational analysis [44,45,46];	In practice, the spark frequency factor changes with varying magnitudes of input process parameters and the suspended powder particle size. Hence, a variable spark frequency factor is used for modelling.
Validation of Existing Models for PMEDM	
The existing model for PMEDM is validated for limited experimental conditions [5,15,16,31,33]	The proposed model is validated by conducting experiments covering wide range of process parameters adopted in the industry.
Outcome Measures Chosen for Study	
Crater, Surface finish, MRR and recast layer thickness	Crater diameter, crater depth, surface crack density, skewness, kurtosis and chemical alteration of machined surface

**Table 2:** Experimental conditions.

Working Conditions	Descriptions
Discharge duration [T_d_] (µs)	10, 50, 100, 150, 300, 500, 750, 1000, 1500, 3000
Peak current [I_p_] (A)	0.5, 3, 6, 9, 12, 15, 18, 21, 24, 27
Gap voltage [V_g_] (V)	20, 40, 60, 80, 100, 120, 140
Duty factor (τ)	6
MoS_2_ Powder (mean size)	Φ40 μm, Φ90 nm
Powder concentration (g/L)	1 g/L
Machining time (min)	10

**Table 3:** Effect of input process parameters on N, N_p_, and K_n_.

					N	N_p_		K_n_ = N_p_/N	
Exp No	T_d_	I_P_	V_g_	τ	EDM	Φ40 µm	Φ90 nm	Φ40 µm	Φ90 nm
Variation of discharge duration									
1	10	6	80	6	11812	11273	14668	0.95	1.24
2	50				6577	6879	6554	1.05	1.00
3	100				3292	3598	3866	1.09	1.17
4	150				2290	2277	2699	0.99	1.18
5	300				1330	1435	1373	1.08	1.03
6	500				918	1116	1096	1.22	1.19
7	750				712	743	728	1.04	1.02
8	1000				553	552	545	1.00	0.99
9	1500				292	369	366	1.26	1.26
10	3000				116	197	193	1.70	1.67
Variation of peak current									
11	300	0.5	80	6	1908	1812	1767	0.95	0.93
12		3			1465	1626	1591	1.11	1.09
13		6			1330	1435	1373	1.08	1.03
14		9			1331	1435	1288	1.08	0.97
15		12			1385	1307	1456	0.94	1.05
16		15			1418	1337	1454	0.94	1.03
17		18			1329	1331	1381	1.00	1.04
18		21			1307	1266	1262	0.97	0.97
19		24			1251	1261	1265	1.01	1.01
20		27			1173	1254	1171	1.07	1.00
Variation of gap voltage									
21	300	6	20	6	1130	1138	1177	1.01	1.04
22			40		1485	1461	1413	0.98	0.95
23			60		1518	1529	1533	1.01	1.01
24			80		1330	1435	1373	1.08	1.03
25			100		1145	1220	1173	1.07	1.02
26			120		932	914	902	0.98	0.97
27			140		702	659	636	0.94	0.91

T_d_—Discharge duration (µs), I_p_—Peak current (A), V_g_—Gap voltage (V), N—Number of pulses per second in EDM (without powder), N_p_—Number of pulses per second in PMEDM, K_n_—Pulse frequency factor.

**Table 4:** Comparison of experimental and simulated results.

	Measured Crater Diameter (SEM)		Simulation Crater Diameter (with 95% PFE)		Measure Crater Depth (SRP)		Simulation Crater Depth (with 45% PFE)		Crater Diameter Error %		Crater Depth Error %	
Exp No.	Φ40 µm	Φ90 nm	Φ40 µm	Φ90 nm	Φ40µm	Φ90 nm	Φ40 µm	Φ90 nm	Φ40 µm	Φ90 nm	Φ40 µm	Φ90 nm
1	82.01	83.46	75.63	80.70	10.49	10.18	10.65	10.52	7.77	3.31	−1.48	−3.36
2	138.28	141.45	142.80	143.80	16.50	19.79	18.30	18.22	−3.27	−1.66	−10.88	7.93
5	294.51	311.72	312.77	290.87	35.98	35.79	36.31	36.14	−6.20	6.69	−0.93	−0.97
6	354.77	374.11	368.92	368.65	30.30	27.17	44.31	44.35	−3.99	1.46	−46.25	−63.20
6 *					27.46	26.24					−61.35	−69.01
7	363.51	382.54	391.48	373.01	37.56	28.66	53.76	54.06	−7.69	−2.67	−43.13	−88.64
7 *					34.62	32.52					−55.28	−66.25
10	752.27	757.32	802.60	802.63	52.64	49.23	93.24	95.04	−6.69	−5.98	−77.14	−93.06
10 *					49.91	45.67					−86.82	−108.09
						Avg. absolute error (%)			3.34	0.19	-	-
11	110.39	113.32	103.07	104.01	16.80	16.79	17.93	17.54	6.63	8.22	−6.76	−4.50
13	294.51	311.72	312.77	290.87	35.98	35.79	36.31	36.14	−6.20	6.69	−0.93	−0.97
14	320.92	363.14	355.08	350.42	37.69	36.91	40.67	36.15	−10.64	3.50	−7.90	2.05
17	394.52	457.52	455.53	446.10	40.01	39.06	39.49	39.80	−15.47	2.50	1.80	−1.89
18	412.49	467.38	476.96	476.37	44.29	39.79	40.34	39.92	−15.63	−1.92	8.93	−0.32
20	513.25	539.36	526.70	523.47	45.58	42.68	40.47	39.68	−2.62	2.95	11.21	7.03
					Avg. absolute error (%)				7.32	3.65	1.06	0.23
21	269.82	273.33	254.11	253.36	17.37	19.74	17.39	18.33	5.82	7.31	−0.11	7.11
22	273.83	278.45	275.66	273.88	28.36	26.73	27.94	26.89	−0.67	1.64	1.46	0.61
23	269.18	296.35	291.83	292.03	31.04	29.86	31.05	31.81	−8.41	1.46	−0.02	−6.55
24	294.51	311.72	312.77	290.87	35.98	35.79	36.31	36.14	−6.20	6.69	−0.93	−0.97
25	297.00	313.21	313.69	311.87	39.09	39.22	39.76	40.14	−5.62	0.43	−1.71	−2.33
26	309.49	324.39	314.15	327.17	39.74	38.98	40.45	40.02	−1.51	−0.85	−1.79	−2.68
					Avg. absolute error (%)				2.76	2.78	0.52	0.80

* Repeated trial.

## Data Availability

Data presented in this study are available in this article.
